# Specifying behavioural and strategy components of de-implementation efforts targeting low-value prescribing practices in secondary health care

**DOI:** 10.1186/s43058-024-00624-6

**Published:** 2024-08-07

**Authors:** Jennifer Dunsmore, Eilidh Duncan, Sara MacLennan, James N’Dow, Steven MacLennan

**Affiliations:** 1 Academic Urology Unit, University of Aberdeen, Aberdeen, U.K.; 2 Health Service Research Unit, University of Aberdeen, Aberdeen, U.K.

**Keywords:** De-implementation, Secondary care, Prescribing, Low-value, Specification, Behavioural target, Strategies, Interventions, Supplementary analysis, Framework

## Abstract

**Background:**

/Aims

De-implementation, including the removal or reduction of unnecessary or inappropriate prescribing, is crucial to ensure patients receive appropriate evidence-based health care. The utilization of de-implementation efforts is contingent on the quality of strategy reporting. To further understand effective ways to de-implement medical practices, specification of behavioural targets and components of de-implementation strategies are required. This paper aims to critically analyse how well the behavioural targets and strategy components, in studies that focused on de-implementing unnecessary or inappropriate prescribing in secondary healthcare settings, were reported.

**Methods:**

A supplementary analysis of studies included in a recently published review of de-implementation studies was conducted. Article text was coded verbatim to two established specification frameworks. Behavioural components were coded deductively to the five elements of the *Action, Actor, Context, Target, Time* (AACTT) framework. Strategy components were mapped to the nine elements of the Proctor’s ‘measuring implementation strategies’ framework.

**Results:**

The behavioural components of low-value prescribing, as coded to the AACTT framework, were generally specified well. However, the *Actor* and *Time* components were often vague or not well reported. Specification of strategy components, as coded to the Proctor framework, were less well reported. Proctor’s *Actor, Action target: specifying targets, Dose* and *Justification* elements were not well reported or varied in the amount of detail offered. We also offer suggestions of additional specifications to make, such as the ‘interactions’ participants have with a strategy.

**Conclusion:**

Specification of behavioural targets and components of de-implementation strategies for prescribing practices can be accommodated by the AACTT and Proctor frameworks when used in conjunction. These essential details are required to understand, replicate and successfully de-implement unnecessary or inappropriate prescribing. In general, standardisation in the reporting quality of these components is required to replicate any de-implementation efforts.

**Trial registration:**

Not registered.

**Supplementary Information:**

The online version contains supplementary material available at 10.1186/s43058-024-00624-6.

Contributions to literature

- De-implementation in health care is required to ensure appropriate and evidence-based practices are used. Research has focused on understanding the effectiveness of de-implementation efforts; however, the information required to replicate and optimise these efforts is lacking.
- A fuller reporting of strategies and target behaviours, using established specification frameworks, aids our understanding of the quality of reporting and where better reporting can be achieved.
- These findings contribute to the growing literature around de-implementation, by highlighting areas of behaviour change strategies and target behaviours that are specified well and areas that are required to be better specified in future de-implementation strategies.

## Introduction

Many complex behavioural interventions, or strategies, are embedded in healthcare to ensure the delivery of high-quality and cost-efficient care practices [1]. Behaviour change strategies which aim to de-implement (i.e. reduce or remove) a behaviour, as opposed to implement one, have gained traction over recent years as an approach to ensure evidence-based health care [2]. However effective contributions to evidence-based practice are contingent on the quality of de-implementation strategy reporting [3].

Insufficient reporting of behaviour change strategies has been a long-standing issue in implementation and de-implementation alike [4, 5]. Extensive research has been undertaken to understand the difference between implementation and de-implementation [6–9] and the unique strategies required for de-implementation [3, 10–12]. However, in order to effectively tailor, replicate or scale up these efforts, full and precise reporting of the distinct strategies and behaviours used in de-implementation are required [13, 14]. Implementation and behaviour change science provides a platform to gauge the quality of behaviour change reporting. Multiple frameworks have been developed to capture the necessary information of how and why strategies were produced [15–17] and the target behaviour of interest, i.e. ‘who’ has to do ‘what’ [18–20].

Specifying behaviour as ‘who’ does ‘what’ and ‘when’ [19], has been further developed to capture important features of health professional behaviour. The *Action,* *Actor, Context, Target, Time* (AACTT) framework [20] consists of five elements to specify a target behaviour: the *Action* (the discrete activity of focus), the *Actor* (the healthcare professional who does the action), *Context* (the environment or situation in which the action happens), *Time* (when the action takes place) and *Target* (who the action regards).

The Proctor framework [17] offers guidance on salient information that should be offered when reporting an implementation (or de-implementation) strategy. This framework offers a ‘How to’ guide to help reproduce the strategy through nine elements: *Name it* (the title of the strategy), *Define it* (description the strategy and content), *Action* (the processes that take place for the strategy to be enacted), *Actor* (who does the action), *Action target* (the strategy targets, including the unit of analysis), *Temporality* (when the strategy is used), *Dose* (the intensity of the strategy), *Implementation outcome affected* (definition and measurement of implementation outcomes) and *Justification* (the reasons for the selection of the strategy and its content).

The application of these frameworks, used for good reporting practice, in combination aids the understanding of the quality of de-implementation strategy and target behaviour reporting.

De-implementation has a valuable role to facilitate evidence-based practice [2, 8] but can only be utilized and improved upon where essential information is offered [3]. This approach ensures a comprehensive analysis of the reporting of de-implementation to understand where improvements in reporting are required. This study aimed to examine how well behavioural targets and the components of de-implementation strategies, addressing inappropriate prescribing in secondary care settings, were reported.

## Methods

### Design

This was a supplementary analysis [21] of 11 randomised control trials included in a recent systematic review which evaluated behaviour change strategies used to de-implement low-value medication prescribing in secondary care [3]. Details about the search strategy, study selection, risk of bias and synthesis of results can be found in the original review. The review reported on the effectiveness, the barriers and facilitators and unintended consequences of de-implementation [3].

### Data extraction

A bespoke data extraction form incorporated the five elements of the AACTT framework [20] and the nine Proctor framework elements [17]. Definitions are summarised in Table 1. The AACTT framework was applied to the target behaviour that the strategy attempted to change. The Proctor framework was applied to the strategies that were delivered. A coding manual was created with definitions for each element of the frameworks and discussed with the research team (ED, SM, SJM) and revised iteratively. Coding suggestions, derived from our coding progress, can be seen in Table 2. To ensure interpretations of framework definitions were systematic, a second coder (ED) double-coded 45% of the studies. Disagreements in coding were resolved by discussion with the research team and definition interpretations were reviewed.

**Table 1:** Definitions for the AACTT and proctor frameworks

Code Label	Code Definition
AACTT Framework	
Actor	The individual or group of individuals who perform (or should/could preform) the Action
Action	A discrete observable behaviour
Context	The physical, emotional or social setting in which the Actor performs (or should/could perform) the Action
Target	The individual or group of individuals for/with/on behalf of whom the Actor performs the Action
Time	The time period and duration that the Actor performs the Action in the Context with/for the Target
**Proctor Framework**	
Name it	Name of the strategy or strategy (if a formal name is not provided the next available description is provided)
Define it	Define the implementation strategy and any discrete components operationally (Classification of strategy components as defined by the EPOC taxonomy)
Actor	Identify who enacts the strategy (i.e., Who provides the strategy)
Action	Use active verb statements to specify the specific actions, steps, or processes that need to be enacted.
Action targets, level of target	Specify organisational level of who the strategy targets
Action targets, conceptual target	Specify targets according to conceptual models of implementation
Identify unit of analysis	Identify unit of analysis for measuring implementation outcomes
Temporality	Specify when the strategy is used
Dose	Specify dosage of implementation strategy
Implementation outcome affected (Primary outcomes)	Identify and measure the implementation outcome(s) likely to be affected by each strategy
Implementation outcome affected (Secondary outcomes)	Identify and measure the implementation outcome(s) likely to be affected by each strategy
Justification	Provide empirical, theoretical, or pragmatic justification for the choice of implementation strategies

**Table 2:** Suggestions for using the AACTT and Proctor Frameworks for specifying de-implementation

Original definitions	Our suggestions/ notes	Illustrative examples from included studies (some quotations have been shortened)
AACTT Framework		
Action A discrete observable behaviour	Specify actions, often identified in relation to the outcomes	"reduce excessive medication dosing for patients with clinically important renal impairment." [22] (Pg. 624, Introduction)
Actor The individual or group of individuals who perform (or should/could) the Action	Specify specific terms to describe exactly who has to change their behaviour (job role, responsibility, the level of actor (team or individual)	"hospital staff at each intervention site (in total 90 nurses, 11 medical officers and 29 clinical officers providing paediatric care" [23] (Pg. 3, The intervention)
Context The physical, emotional or social setting in which the Actor performs (or should/could) the Action	Specify the clinical setting, location and capacity, the country. Consider ‘context’ frameworks to identify relevant areas of context to ensure key areas of influence such as Culture are considered.	"2 tertiary-care academic hospitals in St John’s, Newfoundland, Canada. The Health Sciences Center (346 acute care beds) provides medicine, pediatric and surgery inpatient services, critical care, cardiac surgery, neurosurgery, plastic surgery, burn treatment, obstetrics/gynecology, and acute psychiatry…The metropolitan area of St John’s has a population of 219,000 people (2017)" [24] (pg. 815, Participants).
Target The individual or group of individuals for/with/on behalf of whom the Actor performs the Action	Specify the characteristics of the patients	"adult patients (i.e., patients aged 18 years and older) with renal insufficiency who were being discharged home from the ED" [22] (pg. 624, Materials and Methods)
Time The time period and duration that the Actor performs the Action in the Context with/for the Target	Specify when the action takes place, this could be in relation to a patient (e.g. at the time of admission) or related to the professional (e.g. end of the day)	"Decision support was provided only when a physician in the intervention group attempted to prescribe a targeted inappropriate medication for a patient aged 65 and older who was being discharged from the ED" [25] (Pg. 1389, Methods)
Proctor Framework		
Name it Name the strategy, preferably using language that is consistent with existing literature.	Specify formal descriptive names using established taxonomies	"a validated clinical prediction model (Feverkidstool) was implemented as a decision rule guiding antibiotic prescription" [26] (Pg. 6, Intervention)
Define it Define the implementation strategy and any discrete components operationally	Specify a detailed description of the intervention, use relevant classification taxonomies	The course on geriatric pharmacology was structured in three main areas…The access to and utilization of each teaching module was linked to a self evaluation test and to specific centralized controls. Each module was divided in four sub-modules that each participant completed with specific case reports and questions [27] (Pg. 55, Intervention) (classified to EPOC taxonomy as Interventions targeted at healthcare workers: Educational materials)
The actor Identify who enacts the strategy (e.g., administrators, payers, providers, patients/consumers, advocates, etc.).	Specify who provides or delivers the strategy, including job role, relationship to those using the strategy n.b., the actor for a computer decision support strategy can be specified as the computer and the people who programmed it	"An expert panel of two doctors of pharmacy, two physician information technology experts, three geriatricians, and three emergency physicians participated in the design of the intervention" [25] (pg. 1389, Methods) "computerized clinical decision support system" [28] (Pg. 1, Abstract)
The action Use active verb statements to specify the specific actions, steps, or processes that need to be enacted.	Specify the requirements to set up or deliver the strategy	"Incentive amounts were calculated and communicated quarterly during routine facility visits. . . After communication of the incentive amount earned, facilities submitted a budget to use their incentive allocation and the study team executed the budget…Every effort was made to disburse incentive funds within 4 weeks." [29] (Pg. 4, Incentive intervention)
Action target (1) Specify targets according to conceptual models of Implementation	Our analysis divided this into 2 distinct targets: (1)Specify the level the strategy participants (& their characteristics)	"All physicians in the participating wards" [27] (Pg. 54, Intervention) [individual]
	(2) Conceptual targets	"We based our intervention on the [PRECEDE] model of behavior change. This model emphasizes the inclusion of predisposing strategies (i.e., increasing provider knowledge about acute respiratory tract infection management), reinforcing strategies (i.e., delivering feedback to providers on past patterns of antibiotic prescribing), and enabling strategies (patient education to reduce antibiotic demand)." [30] (Pg. 223, Interventions)
(2) Identify unit of analysis for measuring implementation outcomes	Specify the unit of analysis (and the level of analysis)	"proportion of visits for upper respiratory tract infection and acute bronchitis at which an antibiotic was prescribed" [30] (Pg. 224, Primary Data Analysis) [Patient visit level]
Temporality Specify when the strategy is used	Specify the timing and length of interaction with a strategy	"Reminders were automatically generated and presented on screen when clinicians entered new information or accessed a patient’s EHR." [28] (pg. 3, CDSS and EHR Integration)[Point of care]
Dose Specify dosage of implementation strategy	Specify how much of the strategy is experienced at each interaction	"Incentive amounts were calculated and communicated quarterly during routine facility visits." [29] (Pg. 4, Incentive intervention)
Implementation outcome affected Identify and measure the implementation outcome(s) likely to be affected by each strategy	Specify any outcomes, regardless if it is a implementation outcome	Not an implementation outcome: "We then assessed the effect of TREAT on the management of inpatients in these sites in a cluster randomized controlled trial." [31] (Pg. 1239, Introduction)
Justification Provide empirical, theoretical, or pragmatic justification for the choice of implementation strategies	Specify the theories, models or frameworks that underpin the strategy. It may be helpful to identify theoretical and empirical evidence.	"Considerable evidence from economic theory and research in other clinical areas suggests that adding a package of feedback, nudges, and peer comparisons could dramatically improve prescribing outcomes. Our investigative team previously showed that relatively simple interventions, grounded in behavioral economics and decision science, that leverage accountability and social norms, can reduce unnecessary antibiotic prescribing for acute respiratory infection (ARI) in primary care practices." [32] (Pg. 720, Introduction)
Additional construct identified in this study		
Interactions	Specify the content/tasks of the strategy that those interacting with the strategy should expect AND how they are expected to interact with the strategy	"Every clinician had to finish his/her e-learning program within 1 month from the start of the study in his/her ward." [27] (Pg. 54, Intervention) "Physicians were asked to inspect TREAT’s result interface, but the final choice of antibiotic treatment was theirs." [24] (Pg. 1240, Methods)

### Data analysis

Data was coded deductively to each framework. The verbatim text was extracted to ensure detail was captured. Characteristics of included studies; behaviour targets and strategy components were tabulated. Strategies were classified to the well-established Effective Practice and Organisation of Care (EPOC) taxonomy to allow for comparison [33].

## Results of the review

Study characteristics can be found in Table 3. Reminder strategies were the most common (8/11 studies) [22, 24–28, 31, 34], education materials (4 studies) [24, 26, 30, 32] and Audit and feedback (3 studies) [26, 30, 32] strategies were the next most common. The low-value prescribing practice (i.e. the behavioural target) included inappropriate antibiotics for a range of illnesses (6 studies) [22, 26, 28, 30, 32, 34], and inappropriate drug prescriptions for the treatment of malaria, renal impairment and of older adults (5 studies) [23–25, 27, 31]. Two strategies included content targeting the patient [26, 32].

**Table 3:** Interventions EPOC classification, focus, type and reported effectiveness

Study	Low-value care prescribing focus	EPOC categories of strategies	Type of strategy	Reported as Effective
Daley et al., 2018 [34]	Antibiotics for asymptomatic bacteriuria	Reminders	Single	Yes
Metlay et al., 2007 [32]	Antibiotic use for acute respiratory infections	[1] educational meetings [2] educational materials [3] audit and feedback	Multi-faceted	Yes
Moja et al., 2019 [27]	Prescription medications	Reminders	Single	Yes
Paul et al., 2006 [28]	Empirical antibiotic treatment	Reminders	Single	Yes
Terrell et al., 2009 [31]	Potentially inappropriate medications in older adults	Reminders	Single	Yes
Terrell et al., 2010 [25]	Excessive medication dosing for patients in renal impairment	Reminders	Single	Yes
Menya et al., 2015 [23]	Artemisinin-based combination therapies for suspected malaria	Pay for performance	Single	Yes
Franchi et al., 2016 [24]	Drug prescription in elderly patients	[1] Educational materials [2] Reminders	Multi-faceted	No
Opondo et al., 2011 [30]	Antibiotic use in non-bloody diarrhoea	[1] Inter-professional education [2] Clinical Practice Guidelines [3] Educational materials [4] Monitoring performance of delivery of healthcare [5] Managerial supervision [6] Local opinion leaders [7] Audit and feedback	Multi-faceted	No
van de Maat et al., 2020 [22]	Antibiotic prescription in children with suspected lower respiratory tract infection	Reminders	Single	No
Yadav et al., 2019 [26]	Antibiotic prescribing for Acute respiratory infection	[1] Educational meetings, Reminders, Educational materials [2] Patient-mediated interventions [3] Patient-mediated interventions [4] Local opinion leaders [5] Monitoring the performance of the delivery of healthcare [6] Audit and Feedback	Multi-faceted	No

Eight studies compared their strategies to a usual care control group [22, 23, 25, 27, 28, 31, 32, 34]. Three studies offered a partial or adapted strategy [24, 26, 30]. The reporting of framework elements for control groups can be found in Additional file 1 and 2. Effectiveness results are reported elsewhere [3].

### Specification of behaviour using the AACTT framework

Table 4. shows the AACTT elements reported for each study. Full verbatim coding can be found in Additional File 3. Elements of AACTT; *Action, Context* and *Target* were reported well. The *Action* was reported for all studies, mostly reported as part of the main outcome (e.g., reduce inappropriate prescribing). Contextual information relating to the physical context including the clinical setting, location and the capacity of location were identified in all studies. Studies were conducted in a range of countries and the majority were in high-income countries [22, 24–28, 31, 32, 34]. Studies were set in emergency departments or urgent care units [22, 25, 26, 31, 32], other ward types [24, 27, 28] or whole hospitals [23, 30, 34].

**Table 4.** Reported AACTT framework domains

Study	Actor	Action	Context	Target	Time
**Daley et al., 2018 **[34]	✓	✓	✓	✓	X
**Franchi et al., 2016 **[24]	✓	✓	✓	✓	X
**Menya et al., 2015 **[23]	X	✓	✓	✓	X
**Metlay et al., 2007 **[32]	✓	✓	✓	✓	X
**Moja et al., 2019 **[27]	✓	✓	✓	✓	✓
**Opondo et al., 2011 **[30]	**~**	✓	✓	✓	✓
**Paul et al., 2006 **[28]	✓	✓	✓	✓	✓
**Terrell et al., 2009 **[31]	✓	✓	✓	✓	✓
**Terrell et al., 2010 **[25]	✓	✓	✓	✓	✓
**van de Maat et al., 2020 **[22]	✓	✓	✓	✓	✓
**Yadav et al., 2019 **[26]	✓	✓	✓	✓	X

^*^✓Reported, ~Partially Reported, X Unclear or not reported

The *Target*s in these studies were the patients. Adults [25, 28, 32, 34], children [22, 30], elderly [24, 31] and a mix of children and adult patients [23, 26, 27] were reported. *Actor* and *Time* elements were underreported and are discussed in further detail.

### Reporting of actor

All studies, bar one [23], specified an actor. “Physician” was the most reported type of *Actor* [22, 24, 25, 27, 28, 31, 34], however, other unspecific terms such as “provider” [26] or “clinician” [32] were also reported. Opondo and colleagues (2011) referred to different staff members for four of the seven components of their strategy [30]. Menya and colleagues (2015) did not report an actor, their Pay for Performance incentive strategy was rolled out at the facility level and it was not clear which staff members had to change their behaviour for the incentives to be offered [23].

### Reporting of time

Six (of 11) studies reported the *Time* at which the *Actor* performs the *Action* [22, 25, 27, 28, 30, 31]. The timing of the decision support strategies highlighted when the *Actor* was performing the *Action* (e.g. writing a prescription) [25, 27, 28, 31]. However, education-focused strategies did not specify when the *Action* was performed [24, 26, 32], except Opondo and colleagues (2011) who specified they were trying to change prescribing behaviour when the patients were admitted to the hospital [30].

### Specification of strategy components to the proctor framework

Table 5 summarises the Proctor elements that were reported. Full verbatim coding can be found in Additional File 4. In all 11 studies, the strategies were *named, defined*, and reported as a clear *unit of analysis*. It should be noted that even when elements were reported, there was variation in the type or amount of information provided. For example, it was possible to specify a name for each of the strategies, but this varied from formal programme names (e.g., “MediDSS: Medilogy Decision Support System” as seen in Moja et al., 2019, p. 3), to other strategies being reported more informally (e.g., “decision support” as seen in Daley et al., 2018, p. 184).

**Table 5:** Reported Proctor framework domains

Study	Name it	Define it	Actor	Action	Action target, Level (& characteristics)	Action target, Conceptual targets	Action target, Identify unit of analysis	Temporality	Dose	Primary outcome an implementation outcome?	Justification
**Daley et al., 2018 **[34]	✓	✓	✓	✓	✓(X)	X	✓	~	X	No	✓
**Franchi et al., 2016 **[24]	✓	✓	✓	✓	✓(X)	✓	✓	✓	X	No	✓
**Menya et al., 2015 **[23]	✓	✓	✓	✓	✓(X)	✓	✓	✓	✓	No	✓
**Metlay et al., 2007 **[21]	✓	✓	✓	✓	✓(X)	✓	✓	~	X	No	✓
**Moja et al., 2019 **[27]	✓	✓	✓	✓	✓(X)	✓	✓	✓	✓	No	✓
**Opondo et al., 2011 **[30]	✓	✓	~	~	✓(✓)	✓	✓	~	~	No	X
**Paul et al., 2006 **[28]	✓	✓	✓	✓	✓(X)	X	✓	✓	X	No	X
**Terrell et al., 2009 **[31]	✓	✓	✓	✓	✓(✓)	✓	✓	✓	X	No	✓
**Terrell et al., 2010 **[25]	✓	✓	✓	✓	✓(✓)	X	✓	✓	✓	No	✓
**van de Maat et al., 2020 **[22]	✓	✓	X	✓	✓(X)	X	✓	✓	X	No	✓
**Yadav et al., 2019 **[26]	✓	✓	✓	~	✓(X)	✓	✓	~	~	No	✓

✓Reported, ~Partially Reported, X Unclear or not reported

The steps required to set up the strategy that were identified in the Proctor’s *Action* were generally well reported. Three studies [22, 25, 31] refer to consultation with experts in the design of their strategies. Paul and colleagues (2006) offered more precise details of the information required (i.e., complete patient demographics and test results) for their strategy to produce decision-support output. The *Actor, Action target: specifying targets, Dose* and *Justification* elements were varied or not well reported.

### Reporting of actor

For strategies using decision support, Proctor’s *Actor*, defined as who implements the strategy or the strategy provider, was lacking. Where decision support may be automated and integrated into the electronic health record, a strategy provider is not always applicable or could be identified as the computerised health system [25, 27, 28, 31, 34].

For strategies that used education, *Actors* were better specified, and some studies specified where they sourced their *Actors*. Metlay and colleagues (2007) sourced a clinical leader from each site to host training sessions [32]. Opondo and colleagues (2011) listed *Actors* for three of seven parts in their multifaceted strategy which included a paediatrician from the study team and a local site-based facilitator [30]. Again, the amount of detail provided varied as seen in Menya and colleague's study (2015), where *Actors* were from the "study team" (p. 4) [23].

### Reporting of Action target, specifying targets

Proctor's definition of *Action Target* or “Target(s) of the action” (p.6) has two parts, one part is defined as: “[Identification of a] unit of analysis for measuring implementation Outcomes” (p. 4) and the other as: “where [strategies] are directed or the conceptual ‘targets’ they attempt to impact” (p. 5) [17]. This analysis maintained the definition regarding the unit of analysis. However, extended the definition regarding the ‘conceptual targets’ into the level of participants targeted (e.g. individual, hospital) and relevant participant characteristics, in addition to the identification of conceptual targets (e.g. knowledge, social support). We distinguished the Action Target - level and characteristics element from AACTT’s Actor, by collecting the level the strategy targeted, for example: “All physicians in the participating wards" (Pg. 54) [24], indicates that individuals were the level of action target and "facility-directed" (Pg. 4) [23] meant sites were the level targeted.

All studies reported the *level* of participants the strategy was aiming to target, nine strategies targeted individuals [22, 24–28, 31, 32, 34] and two targeted facilities [23, 30]. Where possible, we also collected relevant *characteristics* of the participants that related to the strategy. Four studies provided a count of clinicians included in the trial [25, 26, 30, 31]. Three studies offered participants’ *characteristics*, Terrell and colleagues (2009) and (2010) captured gender, job status and time since training demographics. Opondo and colleagues (2011) captured gender, age, qualifications and time in their roles.

Seven (of 11) studies reported the *conceptual targets* the strategy attempted to change [23, 24, 26, 27, 30–32] to varying degrees. Franchi and colleagues (2016) reported that their education strategy attempted to: “enhance knowledge and performance” (p. 54) [24]. Whereas Menya and colleagues’ (2015) strategy attempted to: “foster cooperation between departments” (p. 4) [23]. Other studies were less specific, such as Moja and colleagues (2018) who wished to: “encourage better adherence to evidence-based guidelines” (p. 2) [27].

### Reporting of dose

Five (of 11) studies specified the *Dose* for all or some of their strategies [23, 25–27, 30]. The *Dose* was poorly reported across different types of strategies. Two (of eight) strategies using decision support [25, 27], reported the intensity of the decision support, for example: “presented on screen when clinicians entered new information” (Moja et al., 2019, p. 3). Strategies using education components were also poorly reported [24, 26, 30, 32]. Yadav and colleagues (2019) specified a “monthly” dose and Opondo and colleagues (2011) stated a “six-monthly” dose for the audit and feedback strategies, but both failed to specify the dose for their education strategies.

### Reporting of Justification

In total, nine studies (of 11) offered a *Justification* of why a strategy was used [22–27, 31, 32, 34]. One study took a pragmatic approach and did not offer empirical or theoretical reasoning [34]. In multiple cases [27, 34], authors were staff members in the hospital where the strategy was run, which may have informed their approach. This could have been the case for other studies, but this was less clear.

Six studies referenced empirical research only [22–25, 27, 31]. Justifications referred to empirical work as the reason for using the strategy and why the strategy would be suitable to the setting. One study referred to their own previously published work [25].

Only two studies cited established theories to inform their strategy development. Metlay and colleagues (2007) used the Predisposing, Reinforcing and Enabling Constructs in Educational Diagnosis and Evaluation (PRECEDE) model of behaviour change [29]. Metlay and colleagues referenced mechanisms of increasing knowledge, use of feedback and patient education in attempts to reduce antibiotic prescribing [32]. Yadav and colleagues (2019) referred to behavioural economics and decision science, and referenced mechanisms of accountability and social norms [35, 36] that they expected their strategy to impact [26]. Neither of these studies measured these mechanisms, as trial outcomes focused on effectiveness of the strategies.

Franchi and colleagues (2016), did not reference theory but referenced potential mechanisms through which they expected their strategy to work. They postulated that their education strategy would increase knowledge which, in turn, would decrease inappropriate prescribing [24].

#### Additional element identified: interactions

Throughout the analysis, we identified another area of relevance. The way *Actors* (i.e. those that were required to change) engaged or were to engage with the strategies was identified, we have named this element ‘Interactions’. *Interactions* were reported (or partly reported) in seven studies (of 11) [24, 25, 27, 28, 30, 31, 34] (see Table 6). The expected interactions offered details of how the strategy was to be used, for example, “Physicians were asked to inspect [the systems] interface” (Paul et al., 2006, p. 1240) or "The prescriber had the option to order a recommended alternative therapy or to reject the recommendations” (Terrell et al., 2009, p.1389). See Additional file 5 for verbatim coded text.

**Table 6:** Additional identified element of ‘interactions’ reported

Study	Interactions reported
**Daley et al., 2018 **[34]	✓
**Franchi et al., 2016 **[24]	**~**
**Menya et al., 2015 **[23]	X
**Metlay et al., 2007 **[32]	X
**Moja et al., 2019 **[27]	✓
**Opondo et al., 2011 **[30]	**~**
**Paul et al., 2006 **[28]	✓
**Terrell et al., 2009 **[31]	✓
**Terrell et al., 2010 **[25]	✓
**van de Maat et al., 2020 **[22]	X
**Yadav et al., 2019 **[26]	X

✓Reported, ~Partially Reported, X Unclear or not reported

Franchi and colleagues set out their expectations for participants’ interactions with the educational component; "Every clinician had to finish his/her e-learning program within 1 month" (p. 54), however, they did not specify how health care professionals were to interact with the reminder component – which may have contributed to the lack of its uptake [24]. Elsewhere, some information offered was vague and the interaction with the strategy could be inferred, for example, “Reminders…presented …when clinicians entered new information” (Moja et al., 2019, p. 3).

Our suggestions for using the AACTT and Proctor frameworks, with our additional considerations, for specifying de-implementation have been collated for quick reference. See Table 2.

## Discussion (interpretation of the results)

This supplementary analysis aimed to understand the quality of the reporting of the behaviour targets and de-implementation strategies from studies included in a systematic review addressing the de-implementation of low-value prescribing practices in secondary care. Behavioural targets and strategy components were coded to the AACTT and Proctor frameworks respectively, using a deductive approach. These frameworks used in conjunction allowed assessment of how well low-value care behaviours de-implementation strategies were specified. Our analysis highlighted the reported information, particularly in some key elements, to be lacking, varied or brief in detail. We also highlight another potential element of ‘Interactions’, that provided information on how Actors engage with the strategies, which we deem useful to better understand the process of de-implementation.

A key finding in this review was that elements of *Actor* and *Time* in the AACTT framework were underreported or insufficient in detail. Whereas, other elements of the AACTT framework; *Action, Context* and *Target* were more consistently reported. *Actors* were specified using unspecific language (e.g., “Physician” or “Clinician”) which can infer a prescribing role however, does not give any indication of the medical speciality or tenure of the *Actor*. In a complex health system, there are multiple *Actors *who are responsible for multiple patients and conduct many behaviours. The exact specification of the *Actor* has been recognised to be instrumental to understanding healthcare professionals’ behaviours and identifying who is required to change [20]. If an actor is not sufficiently identified, this could lead to inaction in strategy efforts [13]. Additionally, the* timing *of the behaviour requiring change was also lacking, particularly in studies of strategies that were conducted before the point of care. Admission and discharge were often used to measure the outcome of the prescribing rate, but it was not clear if the *Action* (i.e. prescribing behaviour) was happening at this time.

The insufficient reporting of AACTT’s *Actor* and *Time* elements echoes the results of a recent behavioural analysis of hospital-based antimicrobial stewardship strategies [13]. Duncan and colleagues also found *Actor* and *Time* to be underreported or unspecific. It is likely that when reporting a behaviour authors feel these components can be inferred from information about the *Action* or the *Target* of the behaviour. However, failing to offer specific details about who does the behaviour and when it happens remains problematic. *Actors* could refer to multiple types of staff members, who could prescribe at multiple time points. The lack of specification of the target behaviour makes it difficult to understand the low-value prescribing behaviour and the circumstances in which de-implementation strategies must work.

The Proctor framework was less consistently reported compared to the AACTT framework. Proctor’s *Actor, Action target*, *Dose* and *Justification* elements were not well reported. Proctor’s

*Actors*, or those responsible for delivering the strategy, were well-reported in studies that offered training or monitoring. However, they were underreported in studies where the strategy may not have been facilitated by a person (i.e. computerised decision support). The electronic health system was identified as the *Actor* in these cases. Only two of the eight strategies using decision support specified the people involved in strategy design. It can be assumed that integrating a decision support strategy into an electronic health record can be an involved process relying on software developers and IT staff. Failing to specify a strategy provider and those involved in the process of strategy design and delivery can lead to replication issues [17]. It is interesting to note that a few of the study authors were staff members of the locations where strategies were evaluated. This could mean they had tacit knowledge and/or established relationships with key IT contacts, to facilitate integration of their strategy into the electronic health record.

The brevity of information was a common thread throughout the specification of strategies. Proctor and colleagues’ definition of *Action Target (specify targets)* was operationalised as the unit of analysis and the *level and characteristics* of those targeted, with a separate code to identify the *conceptual targets*.

The *level* of participants was identified in each study, but the *characteristics *of participants lacked detail. We distinguished Proctor’s *Action target* (*level and characteristics*) from AACTT’s *Actor* element, as who the strategy targeted, rather than who completes the target behaviour or who was required to change. The characteristics of Proctor’s *Action target*, although not common in this review, can be distinct from the AACTT’s *Actor*. This was illustrated in Opondo and colleagues’ study where strategies were delivered at the hospital level, but individual health staff behaviours were required to change.

Aside from the description of the role of who was targeted (e.g. ‘physician’), only limited *characteristics*, such as age, gender and tenure were identified. Previous de-implementation literature found identifying who may have higher inappropriate prescribing rates and may be less likely to engage with de-implementation strategies, will help pinpoint and tailor strategies to where they are needed [37]. Healthcare professionals who were open to new evidence, younger and had less clinical experience tended to de-implement targeted practices more quickly [37]. Capturing key characteristics of those using the strategies helps gain insight into the circumstances that may contribute to de-implementation and where future efforts need to focus.

The *Justification* for the use of strategies was another area of insufficient reporting. The *Justification* is a valuable point of reporting to understand the reasons for choosing particular strategies and why they are suitable for the context and the behaviour [17]. Generally, empirical evidence and/or pragmatic rationales were offered as sources of justification. However, the reference to theory was sparse and mentioned by only two studies. The application of theory has previously been reported to be limited in strategy development literature [38, 39].

Theoretical justification offers insight into the mechanisms that influence behaviour change. Mechanisms are important in understanding how the strategy developers envisage the strategy changing the intended target behaviour [40]. Mechanisms could be inferred from the type of strategy being offered; however, the reports of strategy content varied. For example, strategies offering education can target various mechanisms such as knowledge or skills, to varying degrees. Multiple frameworks and theories are now available to facilitate de-implementation [8, 41, 42]. Better specification of theories or frameworks is key to understanding the mechanisms that have to change to facilitate de-implementation [17].

Following the analysis stage and research team discussions, an additional element to specify the participants ‘interactions’ with the strategy was identified as useful addition to these frameworks.

The specification of the ‘interactions’ participants must achieve to successfully engage with the strategy is needed. Even where the low-value behaviour and the content of the strategy were specified, there was little information about how healthcare professionals were expected to use the strategy. Strategies require health care professionals to perform behaviour outwith or in addition to their usual routine. Specifying these ‘interactions’ provided explicit details around these additional behaviours, to further understand how the strategy may fit in the workflow or if it is feasible or acceptable to the participants [43, 44].

Another area of reflection was the AACTT element of *Context*. The definition of *Context* is “The physical, emotional or social setting in which the Actor performs (or should/could perform) the Action” (p.5) [20]. This analysis found various levels and types of contexts reported, which meant we collected multiple layers of context including the capacity of setting, location, and the level of setting. Context is a complex and key area of health care improvement as strategy success can be context-dependent [45]. Many efforts have been taken to identify, measure and report all elements of context [46–51]. Extending the scope or amount of information captured in the *Context* element of the AACTT framework could help identify key influences in de-implementation such as Culture, which is often unconsidered [52].

This study utilised the AACTT and Proctor frameworks to specify low-value prescribing practices and related de-implementation strategies. Using two specification frameworks in conjunction allowed a comprehensive assessment of the quality of reporting of the behavioural targets and the strategy components used in de-implementation. These findings highlight the need for standardised reporting of strategies aimed at de-implementing healthcare professional prescribing behaviour. Implementation journal editors and research funders should consider requesting that de-implementation strategies be specified in accordance with the Proctor and AACTT frameworks, as per our suggestions. This would facilitate clear communication of the target behaviour and the strategies, which would allow for a better understanding of the de-implementation process and provide relevant high-quality information required for future replication.

### Strengths and limitations

The strength of this review was its use of established specification frameworks. Utilising the Proctor and AACTT frameworks in conjunction allowed the specification of valuable information relevant to the de-implementation of unnecessary healthcare behaviours. These frameworks were functional and allowed the identification of additional elements that are relevant to de-implementation strategy reporting.

Limitations included the operationalisation of framework definitions and the nature of the analysis. Definitions were discussed with the research team and double coding ensured definitions were coherent and concise. It is possible that another research team could have operationalised elements differently. Additionally, t

he review, this analysis is based on, had a precise inclusion and exclusion criteria, which may have excluded more poorly reported studies, which could have led to an underestimation of suboptimal reporting.

Another limitation was the potential for overlap between the frameworks used. As definitions evolved, we attempted to keep within the definitions of the framework to gain as comprehensive and distinct information as possible. Although not identified often in this analysis, the distinction between the AACTT *Actor* and Proctor’s *Action Target* can be different populations. For example, in the case of patient-mediated strategies, attempts to change healthcare professional's (AACTT *Actor*) behaviour is influenced by targeting patients (Proctor, *Action Target*). Establishing distinct definitions provided guidance for our analysis.

## Conclusions

In conclusion, this analysis provides a better understanding of how well the behavioural targets and the components of de-implementation strategies were reported. The use of AACTT and Proctor’s frameworks in conjunction offers a comprehensive way to specify and report de-implementation research and should be considered by authors, journal editors and funders. The ‘interactions’ of the participants using the strategy and the extension of the AACTT’s *Context* element were identified as additional considerations when reporting de-implementation strategies.

### Supplementary Information

Additional file 1. Identification of the behavioural elements as coded to the AACTT framework domians for control groups.

Additional file 2. Identification of the intervention elements as coded to the Proctor framework domians for control groups.

Additional file 3. Identification of the behavioural elements as coded to the AACTT framework domians for strategy arms.

Additional file 4. Identification of the intervention elements as coded to the Proctor framework domians for strategy arms.

Additional file 5. Identification of the new 'Interactions' component for strategy and control arms.

## Abbreviation

AACTT: Actor, Action Context, Target and Time Framework,

## Declarations

### Availability of data and materials

The data analysed in this analysis is available from the corresponding author on reasonable request.

## References

[1] Grol R, Wensing M, Eccles MP. Improving Patient Care [Internet]. 1st ed. John Wiley & Sons, Ltd; 2013 [cited 2024 Mar 12]. Available from: https://onlinelibrary.wiley.com/doi/10.1002/9781118525975

[2] Grimshaw JM, Patey AM, Kirkham KR, Hall A, Dowling SK, Rodondi N, et al. De-implementing wisely: developing the evidence base to reduce low-value care. BMJ Qual Saf. 2020;29:409–17. 32029572 10.1136/bmjqs-2019-010060 PMC7229903

[3] Dunsmore J, Duncan E, MacLennan S, N’Dow J, MacLennan S. Effectiveness of de-implementation strategies for low-value prescribing in secondary care: a systematic review. Implement Sci Commun. 2023;4:115. 37723589 10.1186/s43058-023-00498-0 PMC10507868

[4] Michie S, Fixsen D, Grimshaw JM, Eccles MP. Specifying and reporting complex behaviour change interventions: the need for a scientific method. Implement Sci. 2009;4:40. 19607700 10.1186/1748-5908-4-40 PMC2717906

[5] Hoffmann TC, Erueti C, Glasziou PP. Poor description of non-pharmacological interventions: analysis of consecutive sample of randomised trials. BMJ. 2013;347:f3755. 24021722 10.1136/bmj.f3755 PMC3768250

[6] Patey AM, Hurt CS, Grimshaw JM, Francis JJ. Changing behaviour ‘more or less’—do theories of behaviour inform strategies for implementation and de-implementation? A critical interpretive synthesis. Implement Sci. 2018;13:134. 30373635 10.1186/s13012-018-0826-6 PMC6206907

[7] Patey AM, Grimshaw JM, Francis JJ. Changing behaviour, ‘more or less’: do implementation and de-implementation interventions include different behaviour change techniques? Implement Sci. 2021;16:20. 33632274 10.1186/s13012-021-01089-0 PMC7905859

[8] Norton WE, Chambers DA. Unpacking the complexities of de-implementing inappropriate health interventions. Implement Sci. 2020;15:2. 31915032 10.1186/s13012-019-0960-9 PMC6950868

[9] Ingvarsson S, Hasson H, von Thiele Schwarz U, Nilsen P, Powell BJ, Lindberg C, et al. Strategies for de-implementation of low-value care—a scoping review. Implement Sci. 2022;17:73. 36303219 10.1186/s13012-022-01247-y PMC9615304

[10] Augustsson H, Ingvarsson S, Nilsen P, von Thiele Schwarz U, Muli I, Dervish J, et al. Determinants for the use and de-implementation of low-value care in health care: a scoping review. Implement Sci Commun. 2021;2:13. 33541443 10.1186/s43058-021-00110-3 PMC7860215

[11] Alishahi Tabriz A, Turner K, Clary A, Hong YR, Nguyen OT, Wei G, et al. De-implementing low-value care in cancer care delivery: a systematic review. Implement Sci. 2022;17:24. 35279182 10.1186/s13012-022-01197-5 PMC8917720

[12] Davey P, Brown E, Charani E, Fenelon L, Gould IM, Holmes A, et al. Interventions to improve antibiotic prescribing practices for hospital inpatients. Cochrane Database Syst. Rev. 2013 [cited 2020 Jun 9];Available from: https://www.cochranelibrary.com/cdsr/doi/10.1002/14651858.CD003543.pub3/abstract 10.1002/14651858.CD003543.pub3 23633313

[13] Duncan EM, Charani E, Clarkson JE, Francis JJ, Gillies K, Grimshaw JM, et al. A behavioural approach to specifying interventions: what insights can be gained for the reporting and implementation of interventions to reduce antibiotic use in hospitals? J Antimicrob Chemother. 2020;75:1338–46. 32016346 10.1093/jac/dkaa001 PMC7177472

[14] Lorencatto F, Charani E, Sevdalis N, Tarrant C, Davey P. Driving sustainable change in antimicrobial prescribing practice: how can social and behavioural sciences help? J Antimicrob Chemother. 2018;73:2613–24. 30020464 10.1093/jac/dky222

[15] Hoffmann TC, Glasziou PP, Boutron I, Milne R, Perera R, Moher D, et al. Better reporting of interventions: template for intervention description and replication (TIDieR) checklist and guide. BMJ. 2014;348:g1687. 24609605 10.1136/bmj.g1687

[16] Ogrinc G, Davies L, Goodman D, Batalden P, Davidoff F, Stevens D. SQUIRE 2.0 (Standards for QUality Improvement Reporting Excellence): revised publication guidelines from a detailed consensus process. BMJ Qual Saf. 2016;25:986–92. 26369893 10.1136/bmjqs-2015-004411 PMC5256233

[17] Proctor EK, Powell BJ, McMillen JC. Implementation strategies: recommendations for specifying and reporting. Implement Sci. 2013;8:139. 24289295 10.1186/1748-5908-8-139 PMC3882890

[18] Fishbein M, Ajzen I. Predicting and Changing Behavior: The Reasoned Action Approach. London, UNITED STATES: Taylor & Francis Group; 2009 [cited 2023 Oct 11]. Available from: http://ebookcentral.proquest.com/lib/abdn/detail.action?docID=668501

[19] Michie S, Johnston M. Changing clinical behaviour by making guidelines specific. BMJ. 2004;328:343–5. 14764503 10.1136/bmj.328.7435.343 PMC338112

[20] Presseau J, McCleary N, Lorencatto F, Patey AM, Grimshaw JM, Francis JJ. Action, actor, context, target, time (AACTT): a framework for specifying behaviour. Implement Sci. 2019;14:102. 31806037 10.1186/s13012-019-0951-x PMC6896730

[21] Heaton J. Secondary analysis of qualitative data: an overview. Hist Soc Res Hist Sozialforschung. 2008;33:33–45.

[22] van de Maat JS, Peeters D, Nieboer D, van Wermeskerken AM, Smit FJ, Noordzij JG, et al. Evaluation of a clinical decision rule to guide antibiotic prescription in children with suspected lower respiratory tract infection in The Netherlands: A stepped-wedge cluster randomised trial. PLOS Med. 2020;17:e1003034. 32004317 10.1371/journal.pmed.1003034 PMC6993966

[23] Menya D, Platt A, Manji I, Sang E, Wafula R, Ren J, et al. Using pay for performance incentives (P4P) to improve management of suspected malaria fevers in rural Kenya: a cluster randomized controlled trial. BMC Med. 2015;13:268. 26472130 10.1186/s12916-015-0497-y PMC4608124

[24] Franchi C, Tettamanti M, Djade CD, Pasina L, Mannucci PM, Onder G, et al. E-learning in order to improve drug prescription for hospitalized older patients: a cluster-randomized controlled study: E-learning to improve drug prescription. Br J Clin Pharmacol. 2016;82:53–63. 26922904 10.1111/bcp.12922 PMC4917810

[25] Terrell KM, Perkins AJ, Hui SL, Callahan CM, Dexter PR, Miller DK. Computerized decision support for medication dosing in renal insufficiency: a randomized controlled trial. Ann Emerg Med. 2010;56:623-629.e2. 20452703 10.1016/j.annemergmed.2010.03.025

[26] Yadav K, Meeker D, Mistry RD, Doctor JN, Fleming-Dutra KE, Fleischman RJ, et al. A Multifaceted Intervention improves prescribing for acute respiratory infection for adults and children in emergency department and urgent care settings. Acad Emerg Med. 2019;26:719–31. 31215721 10.1111/acem.13690 PMC8146207

[27] Moja L, Polo Friz H, Capobussi M, Kwag K, Banzi R, Ruggiero F, et al. Effectiveness of a hospital-based computerized decision support system on clinician recommendations and patient outcomes: a randomized clinical trial. JAMA Netw Open. 2019;2:e1917094. 31825499 10.1001/jamanetworkopen.2019.17094 PMC6991299

[28] Paul M, Andreassen S, Tacconelli E, Nielsen AD, Almanasreh N, Frank U, et al. Improving empirical antibiotic treatment using TREAT, a computerized decision support system: cluster randomized trial. J Antimicrob Chemother. 2006;58:1238–45. 16998208 10.1093/jac/dkl372

[29] Mullen PD, Hersey JC, Iverson DC. Health behavior models compared. Soc Sci Med. 1987;24:973–81. 3616691 10.1016/0277-9536(87)90291-7

[30] Opondo C, Ayieko P, Ntoburi S, Wagai J, Opiyo N, Irimu G, et al. Effect of a multi-faceted quality improvement intervention on inappropriate antibiotic use in children with non-bloody diarrhoea admitted to district hospitals in Kenya. BMC Pediatr. 2011;11:109. 22117602 10.1186/1471-2431-11-109 PMC3314405

[31] Terrell KM, Perkins AJ, Dexter PR, Hui SL, Callahan CM, Miller DK. Computerized decision support to reduce potentially inappropriate prescribing to older emergency department patients: a randomized, controlled trial: decision support for inappropriate prescribing. J Am Geriatr Soc. 2009;57:1388–94. 19549022 10.1111/j.1532-5415.2009.02352.x

[32] Metlay JP, Camargo CA, MacKenzie T, McCulloch C, Maselli J, Levin SK, et al. Cluster-randomized trial to improve antibiotic use for adults with acute respiratory infections treated in emergency departments. Ann Emerg Med. 2007;50:221–30. 17509729 10.1016/j.annemergmed.2007.03.022

[33] Effective Practice and Organisation of Care. Effective Practice and Organisation of Care (EPOC). EPOC Taxonomy. 2015 [cited 2021 Jun 16];Available from: https://epoc.cochrane.org/sites/epoc.cochrane.org/files/public/uploads/taxonomy/epoc_taxonomy.pdf

[34] Daley P, Garcia D, Inayatullah R, Penney C, Boyd S. Modified reporting of positive urine cultures to reduce inappropriate treatment of asymptomatic bacteriuria among nonpregnant, noncatheterized inpatients: a randomized controlled trial. Infect Control Hosp Epidemiol. 2018;39:814–9. 29804552 10.1017/ice.2018.100

[35] Meeker D, Linder JA, Fox CR, Friedberg MW, Persell SD, Goldstein NJ, et al. Effect of behavioral interventions on inappropriate antibiotic prescribing among primary care practices: a randomized clinical trial. JAMA. 2016;315:562–70. 26864410 10.1001/jama.2016.0275 PMC6689234

[36] Persell SD, Doctor JN, Friedberg MW, Meeker D, Friesema E, Cooper A, et al. Behavioral interventions to reduce inappropriate antibiotic prescribing: a randomized pilot trial. BMC Infect Dis. 2016;16:373. 27495917 10.1186/s12879-016-1715-8 PMC4975897

[37] van Bodegom-Vos L, Davidoff F, van de Marang Mheen PJ. Implementation and de-implementation: two sides of the same coin? BMJ Qual Saf. 2017;26:495–501. 27512102 10.1136/bmjqs-2016-005473

[38] Davies P, Walker AE, Grimshaw JM. A systematic review of the use of theory in the design of guideline dissemination and implementation strategies and interpretation of the results of rigorous evaluations. Implement Sci. 2010;5:14. 20181130 10.1186/1748-5908-5-14 PMC2832624

[39] Niven DJ, Mrklas KJ, Holodinsky JK, Straus SE, Hemmelgarn BR, Jeffs LP, et al. Towards understanding the de-adoption of low-value clinical practices: a scoping review. BMC Med. 2015;13:255. 26444862 10.1186/s12916-015-0488-z PMC4596285

[40] Lewis CC, Boyd MR, Walsh-Bailey C, Lyon AR, Beidas R, Mittman B, et al. A systematic review of empirical studies examining mechanisms of implementation in health. Implement Sci. 2020;15:21. 32299461 10.1186/s13012-020-00983-3 PMC7164241

[41] Nilsen P, Ingvarsson S, Hasson H, von Thiele Schwarz U, Augustsson H. Theories, models, and frameworks for de-implementation of low-value care: a scoping review of the literature. Implement Res Pract. 2020;1:2633489520953762. 37089121 10.1177/2633489520953762 PMC9978702

[42] Walsh-Bailey C, Tsai E, Tabak RG, Morshed AB, Norton WE, McKay VR, et al. A scoping review of de-implementation frameworks and models. Implement Sci. 2021;16:100. 34819122 10.1186/s13012-021-01173-5 PMC8611904

[43] Sekhon M, Cartwright M, Francis JJ. Development of a theory-informed questionnaire to assess the acceptability of healthcare interventions. BMC Health Serv Res. 2022;22:279. 35232455 10.1186/s12913-022-07577-3 PMC8887649

[44] Sekhon M, Cartwright M, Francis JJ. Acceptability of healthcare interventions: an overview of reviews and development of a theoretical framework. BMC Health Serv Res. 2017;17:88. 28126032 10.1186/s12913-017-2031-8 PMC5267473

[45] Dixon-Woods M. The problem of context in quality improvement. In: Perspectives on context. London: The Health Foundation; 2014.

[46] Squires JE, Aloisio LD, Grimshaw JM, Bashir K, Dorrance K, Coughlin M, et al. Attributes of context relevant to healthcare professionals’ use of research evidence in clinical practice: a multi-study analysis. Implement Sci. 2019;14:52. 31113449 10.1186/s13012-019-0900-8 PMC6530177

[47] Squires JE, Graham I, Bashir K, Nadalin-Penno L, Lavis J, Francis J, et al. Understanding context: A concept analysis. J Adv Nurs. 2019;75:3448–70. 31359451 10.1111/jan.14165

[48] Pfadenhauer LM, Mozygemba K, Gerhardus A, Hofmann B, Booth A, Lysdahl KB, et al. Context and implementation: A concept analysis towards conceptual maturity. Z Evidenz Fortbild Qual Im Gesundheitswesen. 2015;109:103–14. 10.1016/j.zefq.2015.01.004 26028447

[49] Pfadenhauer LM, Gerhardus A, Mozygemba K, Lysdahl KB, Booth A, Hofmann B, et al. Making sense of complexity in context and implementation: the Context and Implementation of Complex Interventions (CICI) framework. Implement Sci. 2017;12:21. 28202031 10.1186/s13012-017-0552-5 PMC5312531

[50] Rogers L, De Brún A, McAuliffe E. Defining and assessing context in healthcare implementation studies: a systematic review. BMC Health Serv Res. 2020;20:591. 32600396 10.1186/s12913-020-05212-7 PMC7322847

[51] Wells M, Williams B, Treweek S, Coyle J, Taylor J. Intervention description is not enough: evidence from an in-depth multiple case study on the untold role and impact of context in randomised controlled trials of seven complex interventions. Trials. 2012;13:95. 22742939 10.1186/1745-6215-13-95 PMC3475073

[52] Mafi JN, Parchman M. Low-value care: an intractable global problem with no quick fix. BMJ Qual Saf. 2018;27:333–6. 29331955 10.1136/bmjqs-2017-007477 PMC6727656

